# Analysis of lipophilic fluorescent products in blood of Alzheimer's disease patients

**DOI:** 10.1111/jcmm.12824

**Published:** 2016-03-16

**Authors:** Zuzana Chmátalová, Martin Vyhnálek, Jan Laczó, Jakub Hort, Alice Skoumalová

**Affiliations:** ^1^ Department of Medical Chemistry and Clinical Biochemistry2nd Faculty of MedicineCharles UniversityPragueCzech Republic; ^2^ International Clinical Research CenterSt. Anne's University Hospital BrnoBrnoCzech Republic; ^3^ Memory Disorders ClinicDepartment of Neurology2nd Faculty of MedicineCharles University in Prague and Motol University HospitalPragueCzech Republic

**Keywords:** Alzheimer's disease, biomarkers, blood, lipofuscin‐like pigments, oxidative stress

## Abstract

Alzheimer's disease (AD) is a severe neurodegenerative disorder characterized by cognitive decline. Prodromal stage of AD, also called mild cognitive impairment (MCI), especially its amnestic type (aMCI), precedes dementia stage of AD. There are currently no reliable diagnostic biomarkers of AD in the blood. Alzheimer's disease is accompanied by increased oxidative stress in brain, which leads to oxidative damage and accumulation of free radical reaction end‐products. In our study, specific products of lipid peroxidation in the blood of AD patients were studied. Lipophilic extracts of erythrocytes (AD dementia = 19, aMCI = 27, controls = 16) and plasma (AD dementia = 11, aMCI = 17, controls = 16) were analysed by fluorescence spectroscopy. The level of these products is significantly increased in erythrocytes and plasma of AD dementia and aMCI patients *versus* controls. We concluded that oxidative stress end‐products are promising new biomarkers of AD, but further detailed characterisation of these products is needed.

## Introduction

Alzheimer's disease (AD) is the most common cause of dementia in the elderly. The dementia stage of AD is preceded by mild cognitive impairment (MCI), especially its amnestic type (aMCI), a prodromal stage of AD, where patients are still self sufficient as well as by a preclinical stage of AD, where patients have no or little cognitive impairment 1, 2. There is currently no effective disease modifying treatment available, but several drugs have been in development. Recent guidelines suggest the need to initiate newly designed therapy as soon as possible 2. Unfortunately, there is no reliable blood‐derived biomarker for use as a diagnostic tool in AD during the aMCI stage. There are several hypotheses explaining the mechanism of AD aetiology and progression. Oxidative stress theory suggests increased free radical production and damage to the brain tissues as an initial stimulus for the development of AD 3.

Polyunsaturated fatty acids (PUFA) are important components of the phospholipid bilayer membranes of neurons. Their conjugated double bond structure makes them prone to free radical damage. Patients with AD have been shown to have significantly increased lipid peroxidation products such as malondialdehyde (MDA), 4‐hydroxynonenal (HNE) or various isoprostanes in the brain tissue 4. Reactive aldehydes and other lipid radicals produced during lipid peroxidation can react at their origin or diffuse to distant tissues, where they attack lipids or proteins. The result of these chain reactions is the formation of lipid peroxidation end‐products called lipofuscin‐like pigments (LFP). The LFP are fluorescent, and their increased levels have been detected in brain tissues in animal models of AD 5.

While there are clinically used biomarkers in cerebrospinal fluid 6, this approach has several limits 7. Several studies have shown that in patients with neurodegenerative disorders there is an increased permeability of the blood–brain barrier 8. Consequently, reactive compounds from the brain tissue can readily diffuse into the bloodstream and attack PUFA in membranes of erythrocytes or soluble plasma proteins leading to the formation of LFP. *Versus* a spinal tap, blood collection is less invasive, cheaper and more practical. Therefore, finding a reliable blood or plasma marker of incipient AD would be highly favourable.

In previous work, elevated accumulation of LFP in the erythrocytes of AD patients has been shown 9. The aim of this study was to evaluate LFP accumulation in aMCI and dementia because of AD and investigate whether we can use LFP measurements as a biochemical marker for the diagnosis of the initial stages of AD. We analysed erythrocytes and plasma to compare fluorescent characteristics of LFP. We then determined which, is more suitable material for LFP measurements.

## Materials and methods

### Participants

A total of 62 older adults were recruited and followed prospectively with annual examinations at the Memory Clinic at Motol University Hospital in Prague, Czech Republic between 2012 and 2014. The group consisted of patients with mild AD, aMCI and cognitively healthy elderly. Participants with depression (>5 points on the 15‐item Geriatric Depression Scale) 10 were excluded. Participants with major brain pathology that could interfere with cognitive functioning such as cortical infarctions, neoplasm, subdural hematoma and moderate to severe vascular lesions (>1 point on Fazekas scale) 11 were also excluded. Participants with a history of diabetes, impaired glucose tolerance, hyperlipidaemia, stroke, chronic inflammatory diseases and anaemia were not included in this study.

In the mild probable AD group, the participants met the Diagnostic and Statistical Manual of Mental Disorders IV criteria for dementia and the National Institute of Neurological and Communicative Disorders and Stroke and Alzheimer Disease and Related Disorders Association criteria for probable AD without biomarker evidence 12. Patients with dementia had an impairment of memory and another cognitive domain, impaired functional activities and their CDR was 1.0 or higher. All AD patients were on a stable dose of cholinesterase inhibitors for at least 3 months, and their Mini Mental State Examination score (MMSE) was higher or equal to 14.

The aMCI patients met published clinical criteria for MCI including memory complaints reported by a patient or caregiver, evidence of memory dysfunction on neuropsychological testing, generally intact activities of daily living and absence of dementia 1. Memory impairment was established when the patient scored more than 1.5 standard deviation (S.D.) below the mean of age‐ and education‐adjusted norms on any memory test 13. Participants meeting the Diagnostic and Statistical Manual of Mental Disorders IV‐TR criteria for dementia were not included. All participants with aMCI and AD had mild to moderate cortical atrophy.

Controls: The group of cognitively healthy elderly was recruited from the older adults attending University of the Third Age at Charles University in Prague or from relatives of patients of the Memory Clinic, Motol University Hospital in Prague. Participants with memory complaints, history of neurological or psychiatric disease, psychiatric medication usage or abnormal neurological examination including gait or movement difficulties were not included. Participants meeting DSM IV‐TR criteria for dementia, Petersen's criteria for MCI 1 or scoring more than 1.5 S.D. below the age‐ and education‐adjusted norms on neuropsychological examination were not included.

Common biochemical analyses of all participants were performed to exclude possible comorbidities. Particularly, concentration of lipids (total cholesterol, HDL‐cholesterol, LDL‐cholesterol, triacylglycerols), glucose, total protein, C‐reactive protein, vitamin B12, folate and homocysteine were evaluated. All above listed biochemical parameters were in recommended physiological ranges (data not shown).

### Sample collection

Five millilitres of blood from all participants were collected in the morning in tubes coated with K_3_EDTA, centrifuged at 4000 × g for 5 min. immediately after collection and plasma and erythrocyte samples were separated and stored at −80°C immediately to prevent any unwanted processes in samples in terms of further lipid and protein oxidation. All samples were collected, manipulated after collection and stored in the same manner. All participants were informed about the purpose of the study and signed an agreement before providing samples. The Ethics Committee of the University Hospital Motol approved the study.

### LFP extraction

Lipofuscin‐like pigments from erythrocytes and the plasma were extracted into the organic solvents by a modified method according to Goldstein and McDonagh 14. The chloroform phase was taken after centrifugation for fluorescence analysis.

### LFP fluorescence measurement

All of the fluorescence spectra from the chloroform extracts were measured on an AMINCO‐bowman Series 2 spectrofluorimeter. Fluorescence analyses of LFP both in erythrocytes and plasma were based on the measurement of tri‐dimensional fluorescence spectra (3D) and synchronous spectra. LFP represent a mixture of compounds with natural fluorescence as a result of the specific chemical structures. To analyse LFP, complex fluorescence analysis was performed. Firstly, 3D fluorescence measurement was performed for excitation in the range of 250–400 nm and emission in the range of 300–500 nm. This method enables us to identify fluorescence maxima listed in the Table 1. Secondly, synchronous fluorescence spectra were performed to further explore fluorescence characteristics. Synchronous spectra were measured for emission in the range of 250–500 nm with the fixed difference for excitation 25 or 50 nm (SYN25 and SYN50 respectively). These spectra represent diagonal cuts of 3D spectra and reveal particular segments of the complex 3D spectrum. From these spectra, additional fluorescence maxima listed in the Table 1 were identified. The quantitative analysis was based on specific wavelengths corresponding to the fluorescence maxima. The tested maxima are summarized in Table 1.

**Table 1:** Summary of the tested fluorescence maxima of LFP in erythrocytes and plasma for various types of fluorescence analyses

Type of spectrum	Tested fluorescence maxima (nm)					
3D	290/350	330/420	350/420	350/460		
SYN25	315/440	333/358	355/385	395/420	415/440	435/460
SYN50	285/335	310/360	330/380	350/400	360/410	

The fluorescence minimum at 260/480 nm (excitation/emission) was appointed as a reference. The ratio of the fluorescence intensity at the maximum and at the reference was used for comparison of different groups and evaluation of the extent of oxidative damage.

The fluorescence can be influenced by internal quenching, which might be different for individual wavelengths. To exclude the possibility that there are some substances present, which might act as internal quenchers, fluorescence in the samples was measured in undiluted and diluted form. Fluorescence in diluted samples decreased with ration of dilution, which confirms the absence of internal fluorescence quenching (data not shown).

### Statistics

Differences in fluorescence intensities between groups of patients and controls in selected fluorescence maxima were analysed using the statistical programme Statview. anova with a post hoc test and the Bonferroni/Dunn criterion was used to evaluate statistical significance. Results are reported as mean values ± S.D.

## Results

### Characteristics of the participants

Samples from 46 patients and 16 controls were used for fluorescence analyses of LFP in the erythrocytes. For plasma analyses, samples of 42 patients and 16 controls were collected. The tested groups are characterized in Table 2.

**Table 2:** Particpant characteristics

	Participants	Men	Women	Age	Average age (±S.D.)	MMSE (±S.D.)
Erythrocytes						
AD	19	10	9	62–91	76.8 (±7)	19.2 (±3)
aMCI	27	18	9	54–86	70.6 (±9)	25.0 (±4)
Controls	16	5	11	65–82	75.0 (±6)	28.4 (±2)
Plasma						
AD	11	7	4	67–84	74.3 (±5)	19.7 (±3)
aMCI	17	13	4	62–87	71.9 (±8)	25.2 (±3)
Controls	16	7	9	65–84	75.3 (±6)	28.3 (±2)

### LFP fluorescence spectra in erythrocytes

Fluorescent products of lipid peroxidation in erythrocytes of patients with AD, aMCI and age‐matched controls were analysed *via* fluorescence spectroscopy. We first evaluated 3D spectra that give a complex view of all fluorophores present in the sample. There were both qualitative and quantitative differences in 3D between the groups. The most pronounced difference in fluorescence intensity between the groups was observed at 290/350 nm (excitation/emission). At this fluorescence maximum, the biggest peak was found in AD, while lower in aMCI and none in the controls. On the other hand, patients with aMCI had the most significant peak at 350/440 nm (excitation/emission), which was lower in AD and the lowest in controls.

**Figure 1:**
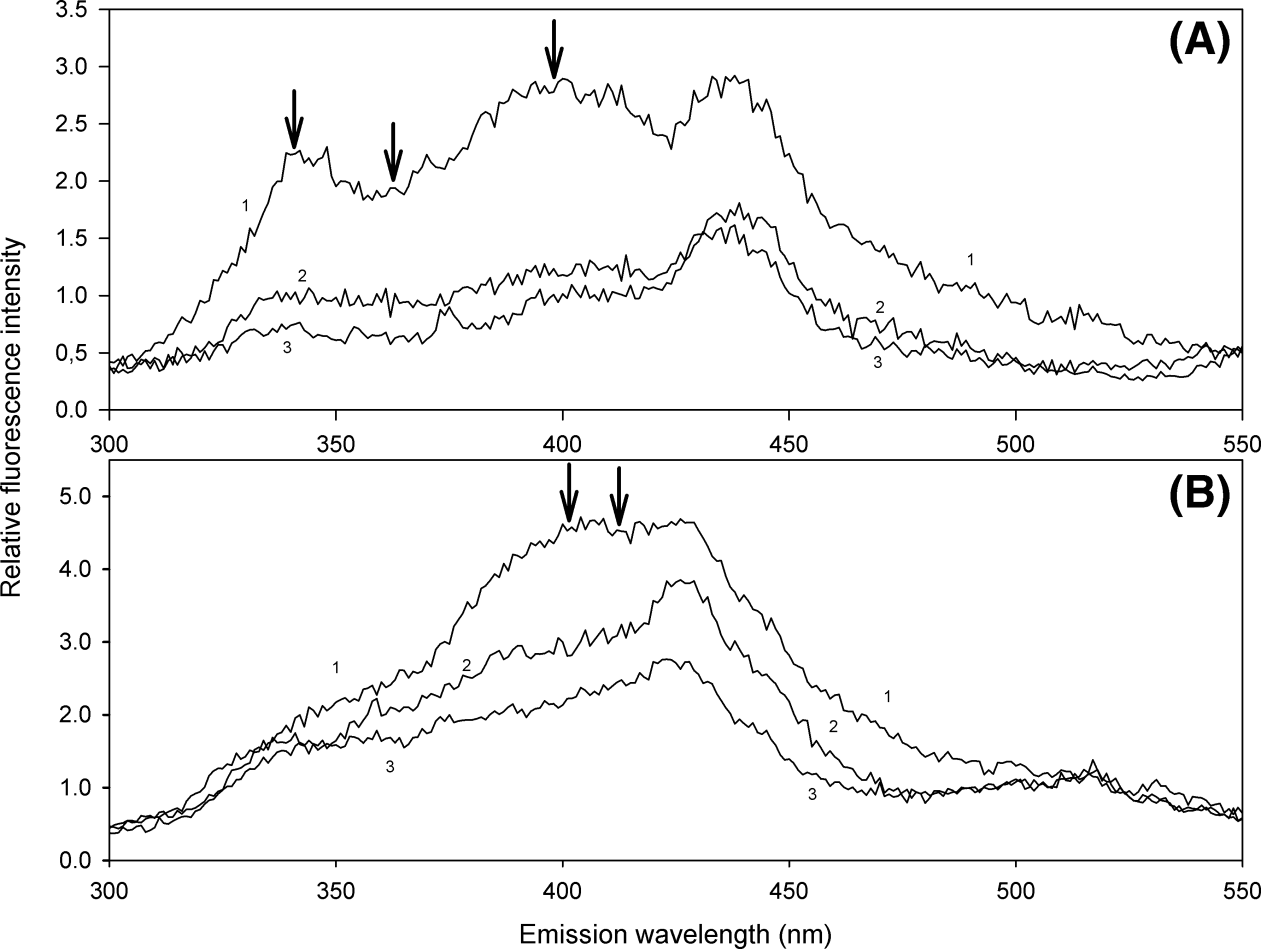
Synchronous spectra SYN50 of LFP in erythrocytes (**A**) and plasma (**B**) of patients with aMCI (1), AD (2) and controls (3). Darts above curves indicate the wavelengths of significant fluorescence maxima.

In synchronous spectra, the fluorescence intensity was different between the groups of patients and controls. Figure 1A shows SYN50. The fluorescence intensity matches the amount of specific fluorophores that were formed in erythrocytes in each group. The highest fluorescence intensity in synchronous spectra was found in patients with aMCI, especially at fluorescence up to 400 nm (emission). Fluorescence intensity in AD was lower compared with aMCI, but still higher than in controls. These differences between the groups were found in both types of synchronous spectra.

### LFP fluorescence spectra in plasma

Fluorescent analyses of plasma LFP extracts were performed similar to the LFP extracts from erythrocytes. Similar to the erythrocytes, there were differences in fluorescence maxima between the groups of patients and controls.

The plasma LFP of patients with aMCI showed the highest fluorescence intensity. The most pronounced differences in fluorescence intensity between patients and controls were detected from 360 to 420 nm (emission) for SYN25 and from 380 to 410 nm (emission) in SYN50 (Fig. 1B).

### Quantitative analyses of LFP in erythrocytes

**Figure 2:**
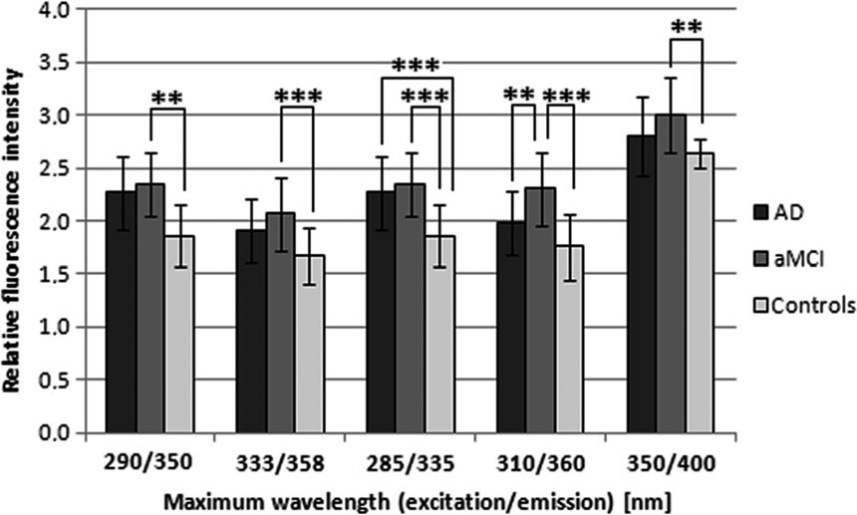
Quantitative analyses of relative fluorescence intensity of LFP in erythrocytes in patients with aMCI, AD and controls at significant fluorescence maxima 290/350 nm, 333/358 nm, 285/335 nm, 310/360 nm and 350/400 nm (excitation/emission). Statistical significance: **P < 0.01 and ***P < 0.001.

The fluorescence maxima found in fluorescence spectra were used for quantitative analyses. Statistically significant differences in fluorescence intensity were found between patients and controls (Fig. 2). In the 3D, the fluorescence intensity was significantly higher in aMCI *versus* controls (118% of controls, *P* = 0.0034) at 290/350 nm (excitation/emission). There was no statistically significant difference between AD and controls.

In the SYN25, a statistically significant difference in fluorescence intensity was found at 333/358 nm (excitation/emission) in aMCI patients *versus* controls (124% of controls, *P* = 0.0004). In the SYN 50, there were three maxima, in which the fluorescence intensity significantly differed between patients and controls. At 285/335 nm (excitation/emission) both aMCI and AD were higher than in controls (ACH: 122% of controls, *P* = 0.0002; aMCI: 126% of controls, *P* < 0.0001). At 310/360 nm (excitation/emission) there were statistical significant differences between aMCI and controls (131% of controls, *P* < 0.0001) as well as between AD and aMCI (117% of AD, *P* = 0.0014) were found. At 350/400 nm (excitation/emission), the fluorescence intensity was different between aMCI and controls (114% of controls, *P* = 0.0016).

### Quantitative analyses of LFP in plasma

The same fluorescence maxima as in quantitative analyses of LFP in erythrocytes were used for determination of LFP in plasma. There was no statistically significant difference between patient groups and controls in 3D fluorescence analysis.

**Figure 3:**
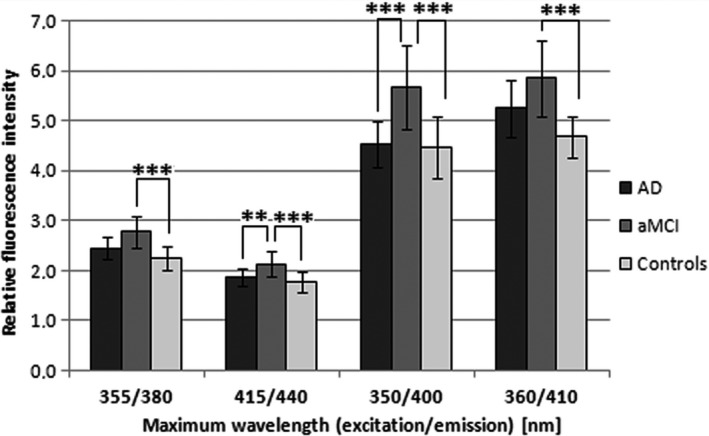
Quantitative analyses of relative fluorescence intensity of LFP in plasma in patients with aMCI, AD and controls at significant fluorescence maxima 355/380 nm, 415/440 nm, 350/400 and 360/410 nm (excitation/emission). Statistical significance: **P < 0.01 and ***P < 0.001.

On the other hand, two fluorescence maxima were found to be different in SYN25 (Fig. 3). At 355/380 nm (excitation/emission), the fluorescence intensity was significantly higher for aMCI *versus* controls (123% of controls, *P* < 0.0001). Moreover, the aMCI group showed significantly higher fluorescence intensity at 415/440 nm (excitation and emission) compared to controls (120% of controls, *P* < 0.0001) and AD patients (115% of AD, *P* = 0.0028).

Finally, in SYN50 spectra, there were two fluorescence maxima with statistically significant differences (Fig. 3). At 350/440 nm (excitation/emission), there was difference in fluorescence intensity between aMCI and controls (127%, *P* < 0.0001) as well as between aMCI and AD patients (125% of ACH, *P* = 0.0002). At 360/410 nm (excitation/emission), a difference was found between aMCI and controls (125% of controls, *P* < 0.0001).

### The comparison of fluorescence analyses of LFP from erythrocytes and plasma

We compared the fluorescence for LFPs in both erythrocytes and plasma to evaluate which biological material is more sensitive for the detection of differences in the amount of end‐products of lipid peroxidation between patients and controls.

The fluorescence was higher in the LFP from plasma extracts than in erythrocytes. However, it does not lead to a better sensitivity for finding differences between patients and controls. More fluorescence signal with statistically significant differences between patients and controls were identified in fluorescence analyses of LFP in erythrocytes. These were at 290/350, 333/358, 285/335, 310/360 and 350/400. On the other hand, for the plasma LFP, the fluorescent maxima were different from these for erythrocytes and are at 355/380, 415/440, 350/400 and 360/410.

## Discussion

Our results show that the LFP formation during the development of AD is measurable both in erythrocytes and plasma and that it is most pronounced in early stages of the disease. Increased oxidative stress, which is not restricted only to the brain tissue 15, and free radical damage has been shown to accompany the onset and progression of AD. Elevated markers of free radical damage to tissues such as MDA, HNE or isoprostanes were detected in whole blood as well as in plasma or urine 4, 16. Erythrocyte membranes contain PUFA, which are very prone to free radical damage. Furthermore, erythrocytes do not have sufficient mechanisms to repair oxidized lipids in their membranes, which leads to accumulation of these products in membranes as well as inside the cell during the erythrocyte lifespan. The free radical chain reactions of lipids and further attack of proteins lead to the formation of unique advanced products of lipid peroxidation – the called LFP. These LFPs are characterized by native fluorescence because of their chemical composition with a complex cyclic structure and conjugated doubled bonds 17. Elevated levels of LFP were previously found in erythrocytes of dogs with canine counterpart of AD 5 as well as in erythrocytes of AD patients with the 27% increase 9. In our study, several significant fluorescent maxima were identified *via* fluorescence analyses of LFPs in erythrocytes the same as in plasma. The amount of LFP in erythrocytes in aMCI was higher by an average of 22% *versus* controls and in AD patients it was 23% *versus* controls. The mean LFP levels in plasma were higher by 24% in aMCI patients than in controls.

We showed here that the fluorescence spectra of LFP in erythrocytes and plasma differ mainly in fluorescence intensity, that is, the quantity of specific fluorophores. Moreover, the results show that the most pronounced fluorescence maxima can be found during the initial stages of AD (in aMCI), which concurs with previous studies 18. Furthermore, our study demonstrates that there are also qualitative differences in the fluorescence spectra with different fluorescence maxima between the groups of patient and controls. This finding indicates that disease progression is accompanied by qualitative changes in specific fluorophores. The composition of LFP during the course of disease progression may reflect the decreasing pool of biomolecules susceptible to free radical damage, decreasing dynamics of reactive compounds formation and different mechanisms of end‐product formation. This phenomenon has been studied and described in detail previously 19.

It is interesting that the significant fluorescence maxima differed not only between the patients and controls but also between LFP extracts of erythrocytes and plasma. This shows that the LFP formation is a dynamic process and demonstrates the uniqueness of LFP. We suppose that the plasma LFP contains more oxidized proteins while the LFP from erythrocytes involve higher portions of oxidized membrane lipids, which results in different fluorescent characteristics. Moreover, small molecular weight lipophilic peptides might appear in the chloroform extracts from plasma and influence their fluorescence characteristics. This can also explain observed differences in fluorescence of erythrocytes and plasm extracts. Although more pronounced fluorescence intensity was measured in the plasma LFP than in erythrocytes, we cannot conclude that the use of plasma for the LFP measurement is preferable. The higher fluorescence intensity and hence higher content of LFP in plasma does not imply higher sensitivity of the fluorescence method for distinguishing between patients and controls. Although some LFP that is present in the erythrocyte membrane does not exhibit as such fluorescence as that in plasma, the erythrocyte samples have higher discriminative potential between patients and controls.

With respect to different amount of LFP in patient groups and controls, LFP determination could be used as an additive marker in early AD diagnostics. Moreover, fluorescence maxima found in both biological materials can be used to differentiate patients in different AD stages from controls. Our results indicate that it is not possible to decide whether erythrocytes or plasma are more appropriate biological materials for LFP determinations. Because of different patterns of fluorescent maxima in plasma and erythrocytes it seems that simultaneous analysis of LFP in the erythrocyte membrane and plasma could be used to increase sensitivity and specificity.

There are several limitations in our study. To definitively prove LFP as a reliable marker for AD diagnosis, it is necessary to conduct more research. Chronic systemic diseases, other neurodegenerative diseases than AD and stroke might be accompanied by oxidative stress and increased end‐products of radical reactions in blood. However, the advantage of using LFP as markers is that their fluorescence characteristics reflecting their composition are specific for various pathologies and can be used as a fingerprint for a particular disease. Nevertheless, it must be further investigated whether the LFP detected in our study are specific for AD. In this study, patients with systemic diseases accompanied by oxidative stress, such as diabetes and hyperlipidaemia, and patients with other neurodegenerative diseases and stroke were not included. In further studies, to find specific biomarkers for AD, LFP from patients with other neurodegenerative diseases and systemic diseases will be tested and matched with our results. Moreover, to validate these found markers as additional diagnostic tools, it will be important to perform measurement in a larger patient population and correlate the findings with neuropsychological and volumetric measurements. Finally, LFP composition must be determined by HPLC and mass spectrometry. We will then propose a concrete mechanism for LFP formation, which could help to better understand AD pathogenesis and develop AD treatments.

## Disclosure

The authors confirm that there are no actual or potential conflicts of interest.
